# Visual Working Memory Capacity for Color Is Independent of Representation Resolution

**DOI:** 10.1371/journal.pone.0091681

**Published:** 2014-03-11

**Authors:** Chaoxiong Ye, Lingcong Zhang, Taosheng Liu, Hong Li, Qiang Liu

**Affiliations:** 1 Department of Educational Science and Technology, Minnan Normal University, Zhangzhou, China; 2 Department of Psychology and Neuroscience Program, Michigan State University, East Lansing, Michigan, United States of America; 3 School of Psychology, Liaoning Normal University, Dalian, China; CSIC-Univ Miguel Hernandez, Spain

## Abstract

**Background:**

The relationship between visual working memory (VWM) capacity and resolution of representation have been extensively investigated. Several recent ERP studies using orientation (or arrow) stimuli suggest that there is an inverse relationship between VWM capacity and representation resolution. However, different results have been obtained in studies using color stimuli. This could be due to important differences in the experimental paradigms used in previous studies.

**Methodology/Principal Findings:**

We examined whether the same relationship between capacity and resolution holds for color information. Participants performed a color change detection task while their electroencephalography was recorded. We manipulated representation resolution by asking participants to detect either a salient change (low-resolution) or a subtle change (high-resolution) in color. We used an ERP component known as contralateral delay activity (CDA) to index the amount of information maintained in VWM. The result demonstrated the same pattern for both low- and high-resolution conditions, with no difference between conditions.

**Conclusions/Significance:**

This result suggests that VWM always represents a fixed number of approximately 3–4 colors regardless of the resolution of representation.

## Introduction

Visual working memory (VWM) is one of the most prominent mechanisms in human information processing system [1], [2]. It provides an online storage for visual information transferred from perceptual processing, which enables us to get a coherent understanding of the visual scene, and it also provides a temporary storage buffer for facilitating extended tasks in daily life [3]–[5]. Due to its significance, it has received much attention from multiple research fields in the last decade [6]–[10].

A central issue in this research concerns the capacity of VWM, that is, how much information can be maintained in VWM at one time. It is generally accepted that VWM capacity is quite limited, perhaps to 3–4 object representations [11]–[16]. Another related issue concerns the resolution of VWM representation, that is, how detailed and precise the memory is with respect to the visual object.

Many behavioral studies have investigated the relationship between VWM capacity and representation resolution [17]–[21]. The majority of these studies have adopted the change detection task, which requires participants to detect whether or not an object is changed between a memory and a probe array [15], [20], [22], [23]. However, there is a potential problem with the change detection task, in that it involves many processing stages other than storage and maintenance, such as encoding, retrieval or comparison (between the memory and probe arrays). These extra stages of processing could all potentially contribute to the behavioral outcome, thus contaminating the measure of VWM storage and maintenance [24].

One technique to on-line track VWM maintenance without the potential contamination is through the use of event-related potentials (ERP) during the retention interval of a memory task. By these means, researchers have identified an ERP component, contralateral delay activity (CDA) during the retention interval [25], whose amplitude is linked directly with the number of representations maintained in VWM [26]–[34]. CDA is a large negative wave which is observed over posterior electrode sites contralateral to the locations of the stored objects. It persists throughout the memory retention interval in the change detection task, and is strongly modulated by the number of representations in VWM but reaches an asymptote once capacity is exhausted.

Several ERP studies using the CDA to index memory storage have found an inverse relationship between VWM capacity and representation resolution. For example, Gao et al. [35] found that, when participants maintained low-resolution visual information (e.g., simple basic shapes), the CDA amplitude for 4 objects was higher than that for 2 objects. However, when participants maintained high-resolution visual information (e.g., complex random polygons), the CDA amplitude was equivalent for 2 and 4 objects. This result suggested that VWM capacity was reduced from 4 to 2 as the representation resolution increased. However, in Gao et al.’s study [35], the complexity of the stimulus and the resolution of the representation co-varied. Thus it is possible that their results were partly due to stimulus complexity instead of the resolution of memory representation. In a further study, Machizawa et al. [36] avoided this potential confound by using the same set of orientation stimuli and manipulated representation resolution by modulating the amount of orientation change between the memory and probe stimuli. They obtained similar findings as Gao et al. [35]: CDA amplitude was higher for 4 than for 2 orientations in the low-resolution condition, but they were equivalent in the high-resolution condition. These results implied that VWM could maintain 1–2 high-resolution representations or 3–4 low-resolution representations, even when both conditions were based on the same visual stimuli(see also ref [37]).

The inverse relationship between VWM capacity and representation resolution has been demonstrated in ERP studies for several visual features, including polygon, Landolt ring, oriented bars and arrows. However, whether this same relationship holds for the color feature is not clear. Color is an important feature for many aspects of visual processing. In addition, color is also widely used in many studies of VWM capacity and resolution [11], [13], [17], [19], [26], [28], [38]–[41]. Indeed, two previous ERP studies do not support the inverse relationship between VWM capacity and representation resolution for color feature. In one study, Ikkai et al. [39] compared CDA for high-contrast and low-contrast color stimuli in a change detection paradigm. Changes in the high-contrast condition was large while change in the low-contrast condition was subtle, thus presumably relying on low- and high-resolution representations, respectively. They found no difference in CDA amplitude across the contrast conditions, but 4 colors evoked a higher CDA than 2 colors. Similarly, Luria et al. [41] used high-similarity and low-similarity color stimuli and found equivalent CDA amplitudes across the high- and low-similarity conditions.

Although these results suggest that capacity and representation resolution do not have an inverse relationship for the color feature, there were two potential problems with these experiment [39], [41]. First, in these studies, the high- and low-contrast (or high- and low- similarity) conditions were based on different visual stimuli. It is known that CDA amplitudes could be different for different stimulus material (orientation and color) with the same number of items [29]. Thus, caution is needed when comparing CDA amplitudes between different colored stimuli. Second, the asymptote of CDA amplitude is a good index to measure the limit of the VWM capacity. In Ikkai et al.’s study [39], they used set sizes of 2 and 4, and in Luria et al.’s study [41], they used set sizes of 2, 4, 6. Although both studies demonstrated that the CDA amplitude for set size 4 was higher than set size 2, it would be possible that the asymptote of CDA amplitude was different between conditions. Such as, in high-contrast (or low-similarity) condition, the CDA amplitude reached an asymptote in 4 items, but, in low-contrast (or high-similarity) condition, it reached an asymptote in 3 items. Here, we wanted to corroborate and extend previous results, while avoiding the above potential problems, so as to further examine the relationship between VWM capacity and representation resolution for the color feature.

In this study, we used a change detection task typically used in ERP research [36], [37], [39], [41], and measured CDA to index the amount of information stored in VWM. We used colored squares as stimuli and manipulated representation resolution by varying the magnitude of color changes between the memory array and the probe. In the high-resolution condition, the changed probe had a similar color to the stored color. In contrast, in the low-resolution condition, the change between probe color and stored color was highly discriminable. By comparing behavioral performance and ERPs in the low- vs. high-resolution condition, we examined the relationship between VWM capacity and representation resolution for color.

## Methods

### Participants

Eighteen right-handed students (12 females) from Liaoning Normal University volunteered to participate in this experiment for pay. They reported no history of neurological problems, reported having normal color vision and normal or corrected-to-normal visual acuity. Written informed consent was provided by each participant prior to the experiment. This research was approved by the Research Ethics Committee of Liaoning Normal University of China and was conducted in accordance with the Declaration of Helsinki.

### Stimuli

Each memory item was a square (size: 0.65°×0.65°) whose color was chosen randomly without replacement from a set of six highly discriminable colors. RGB values for the six colors were: red [233, 0, 0], green [30, 138, 18], blue [26, 49, 178], orange [210, 85, 7], yellow [231, 228, 66], purple [156, 0, 158]. We refer to these six colors as the *original set*. The probe colors in the high-resolution condition consisted of six colors similar to original set: red [216, 0, 0], green [49, 151, 34], blue [50, 60, 192], orange [226, 93, 25], yellow [216, 214, 50], purple [171, 31, 182]. We refer to these six colors as the *similar set*.

### Experiment Design and Procedure

Participants were seated in an electrically shielded and sound-attenuated recording chamber at a distance of 70 cm from a flat cathode ray tube monitor. Items were presented within 4°×7.3° rectangular regions bilaterally, centered 3° to the left and right of the center of the screen. The memory array consisted of 2, 3, or 4 different colored squares in each hemifield with their colors randomly sampled from a set of 6 possible colors (the original set) with the constraint that any given color would not appear more than once in each hemifield. Stimulus positions were randomized on each trial, with the constraint that the distance between squares within a hemifield was at least 2° (center to center).

**Figure 1:**
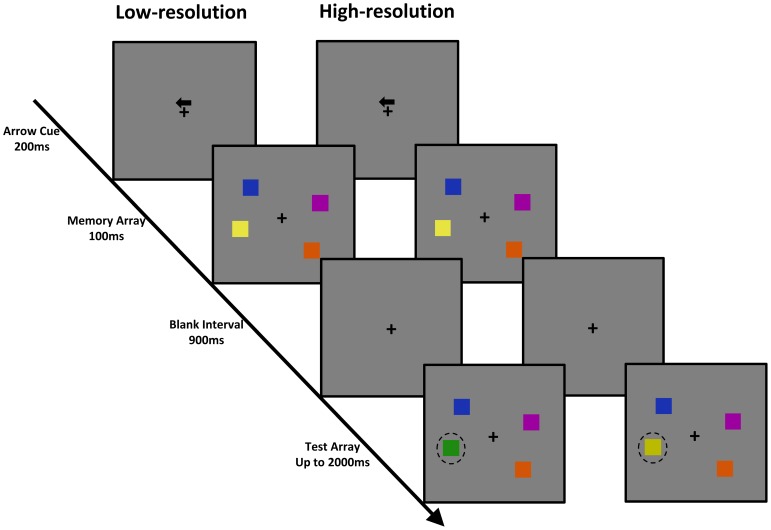
Trial schematic. A low-resolution condition (left) and a high-resolution condition (right) were illustrated. The dashed circle was used to mark the changed item, which was not presented during the experiment.

Each trial began with a 200 ms arrow cue above a fixation point, followed by a 100 ms memory array, a 900 ms blank period and finally, a 2000 ms probe array (Figure 1). Participants were required to keep their eyes fixated on the fixation while storing the colors in the hemifield indicated by the cue. The probe array in the cued hemifield had one different color than the memory array on 50% of trials; they were identical in the remaining trials. The participant’s task was to indicate whether the probe array was identical to the memory array, or a color has changed in the corresponding location between the memory and probe array. We emphasized response accuracy rather than response speed in the instruction. We kept the memory array constant while varying the magnitude of color change between the memory and probe array to manipulate representation resolution. In the low-resolution condition, when a color change occurred, a new color square that was not used in the memory array would be randomly selected from the original set. In the high-resolution condition, a color change would entail presenting a corresponding color from the similar set (e.g., a yellow changed to a different shade of yellow, see Figure 1).

We used a 2 (resolution: high vs. low)×3 (set size: 2 vs.3 vs. 4) within-subject design. The two resolution conditions were blocked and the order of blocks was counterbalanced across participants. There were 200 trials for each set size, with a total of 600 trials per resolution block which were fully randomized. Each block was split into 6 mini-blocks of 100 trials each, with a break of at least 30 s between mini-blocks and 5 min between blocks. The entire experiment lasted approximately 60 min. Before each block, there were at least 20 practice trials to ensure the participants understood the instructions.

### Electroencephalography Recording and Analyses

Electroencephalographic (EEG) activity was recorded from 64 tin electrodes mounted in an elastic cap, using the International 10/20 System. Vertical electrooculogram (VEOG) and horizontal electrooculogram (HEOG) were recorded with two electrodes, one placed below the left eye, and another placed next to the right eye. Impedance at each electrode site was maintained below 5 kΩ. The EEG and EOG were amplified by a QuickAmp amplifier (Brain Products GmbH, Munich, Germany) with a 50 Hz low-pass and were digitized at a sampling rate of 500 Hz.

The EEG was algebraically re-referenced offline to the average of the left and right mastoids during post-recording analyses and segmented into 1100 ms epochs starting from 100 ms before the memory array onset. EEG contaminated with horizontal eye movements greater than 2° (>32 µV HEOG amplitude) were excluded from analysis. Trials with remaining artifacts exceeding ±75 µV in amplitude were rejected. Participants with trial rejection rates that exceeded 25% were excluded from the analyses. Four participants (3 females) were excluded on this basis, such that the results reported below is based on data from 14 participants.

Three pairs of electrode at posterior parietal sites (CP3/CP4, CP5/CP6, and P7/P8) were chosen for analysis based on previous work on CDA [28], [35]–[37]. The contralateral waveforms were computed by averaging the activity recorded at left hemisphere electrode sites when participants were cued to remember the right side of the memory array with the activity recorded from the right hemisphere electrode sites when they were cued to remember the left side. The ipsilateral waveforms were computed by averaging left and right hemisphere sites when participants were cued to remember the left and right side of the memory array, respectively. The CDA was defined by subtracting the ipsilateral activity from the contralateral activity, with a measurement window of 300–900 ms after the onset of the memory array. Following previous work [35], [37], the average CDA waveforms were smoothed by applying a 17 Hz low-pass filter, without loss of relevant information, given that the CDA is a sustained low-frequency wave.

## Results

### Behavioral Data

**Figure 2:**
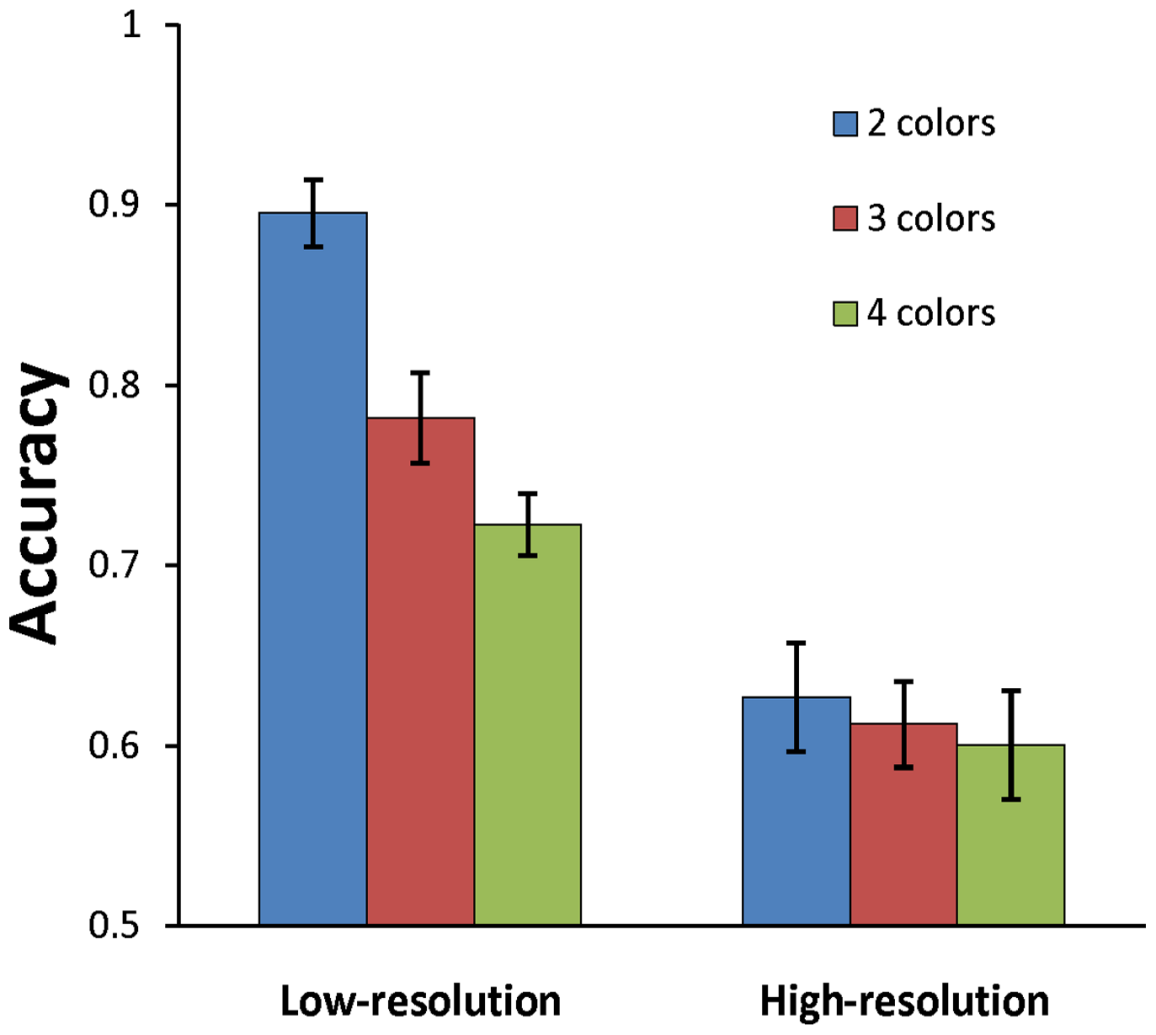
The behavioral results. Error bars show standard error of the mean.

As shown in Figure 2, consistent with the previous findings [39], [41], the accuracy was lower for the high-resolution than for the low-resolution conditions. A two-way ANOVA with set size (2, 3, 4 objects) and resolution condition (low, high) as factors yielded main effects of set size, *F*(2, 26) = 51.002, *p*<.001, and condition, *F*(1, 13) = 94.037, *p*<.001. The interaction between the two factors was also significant, *F*(2, 26) = 28.429, *p*<.001. Separate one-way ANOVAs confirmed that the main effect of set size was only present in the low-resolution condition, *F*(2, 26) = 66.603, *p*<.001, but not in high-resolution condition, *F*(2, 26) = 2.135, *p* = .14. Thus behavioral results suggest that the capacity of VWM suffered a marked reduction in the high-resolution condition.

### Electrophysiological Data

**Figure 3:**
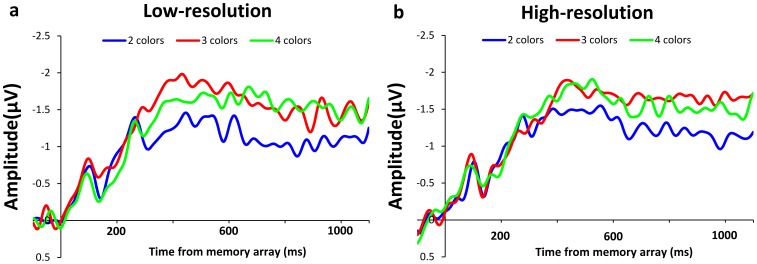
Difference waves of CDA for arrays of 2, 3 and 4 colors in low-resolution (a) and high-resolution (b) conditions. Grand-averaged ERP waveforms were time-locked to the onset of the memory array.

**Figure 4:**
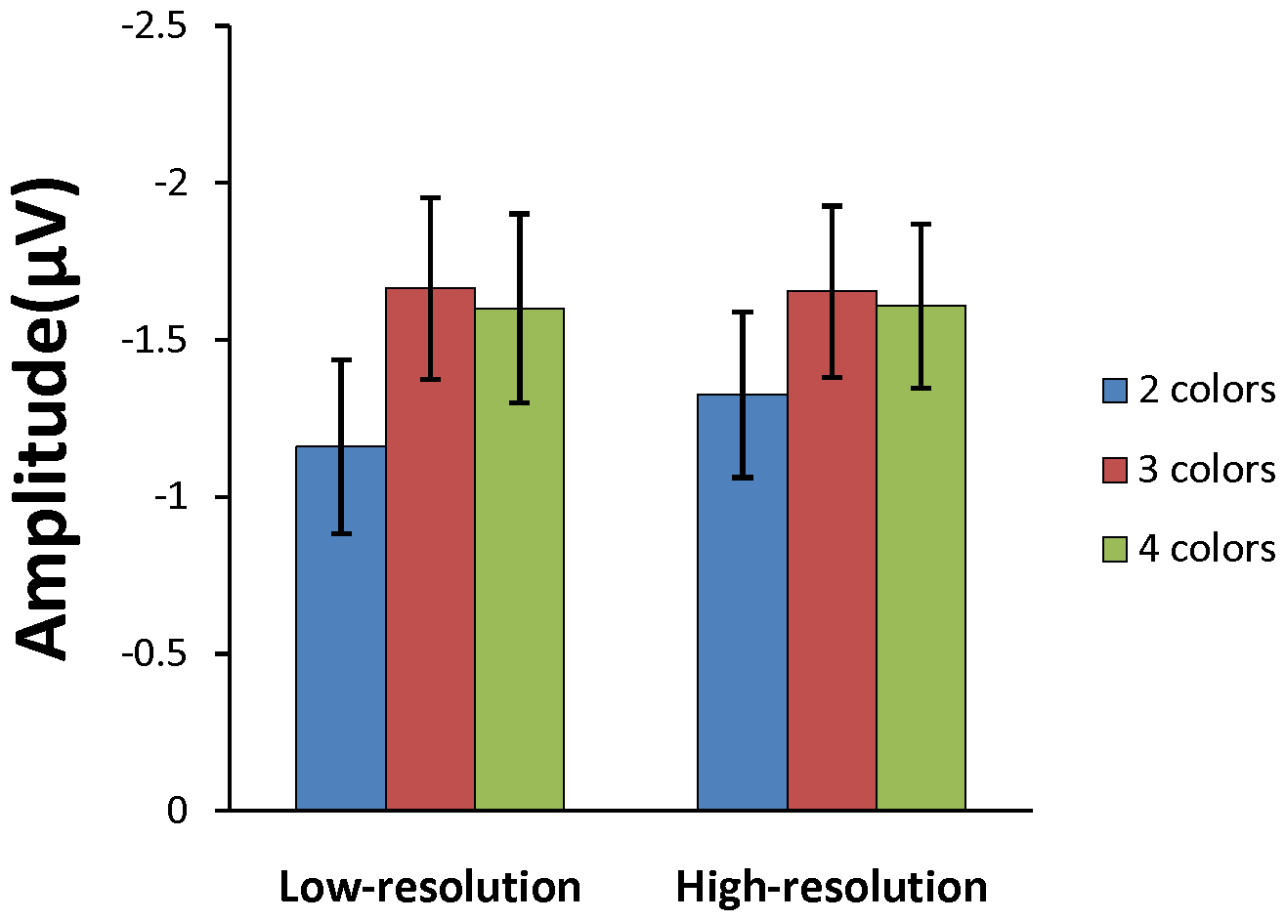
Mean amplitudes of CDA for the low- and high-resolution condition. Error bars show standard error of the mean.

Consistent with prior research [26]–[37], [39], [41], we observed a CDA that emerged around 300 ms after memory array onset and persisted throughout the retention period. The grand average subtraction waveforms for each set size are shown in Figure 3a for the low-resolution condition and in Figure 3b for the high-resolution condition. Figure 4 shows the mean amplitudes of CDA in both conditions. The CDA amplitudes (for each subject and each condition) were submitted to a two-way ANOVA with set size (2, 3, 4 objects) and resolution condition (low, high) as factors. The only significant effect was that of set size, *F*(2, 26) = 7.445, *p*<.01, which reflected an increase in CDA amplitude as more colors were stored. Post-hoc comparisons showed significant difference in CDA amplitude between 2 and 3 colors in low-resolution, *t*(13) = 2.756, *p*<.05, and high-resolution, *t*(13) = 2.892, *p*<.05, condition, while the amplitude was not significantly different between 3 and 4 colors in either low-resolution, *t*(13) = −0.394, *p* = .70, or high-resolution, *t*(13) = −0.366, *p* = .72, condition. This result suggested that participants maintained approximately 3 colors in both resolution conditions. Importantly, neither the main effect of condition, *F*(1, 13) = 0.247, *p* = .63, nor the interaction between condition and set size, *F*(2, 26) = 0.510, *p* = .60, were significant.

## Discussion

The goal of the present experiment is to examine the relationship between VWM capacity and representation resolution for the color feature. To that end, we employed a change detection task with ERP recordings and used the CDA component to index memory storage. Behavioral performance showed a large difference in overall accuracy across the two resolution conditions, demonstrating that our manipulation of resolution demand was effective. However, we observed equivalent CDA amplitude in the low- and high-resolution condition, suggesting that the CDA was not influenced by task difficulty in general. Regardless of the resolution demand of the task, the CDA amplitude for 4 colors was higher than 2 colors but not different from 3 colors. This suggests that VWM always holds a fixed capacity of approximately 3 color representations in both low- and high-resolution conditions. Our results were consistent with Ikkai et al. [39] and Luria et al.’s [41] studies. More importantly, we found that for same memory stimuli, although behavioral accuracy was impaired when high-resolution representations were required, the amplitude and asymptote of the CDA did not change with the need for higher resolution.

Several previous studies have shown that CDA amplitude was not only modulated by the number but also by the encoding level of representation resolution of the memory materials [36], [37]. Our results, on the other hand, support the notion that CDA amplitude reflects only the number of items retained in working memory [28], [39], because CDA amplitudes were no different in the low- and high-resolution conditions and reached an asymptote at 3 items in both conditions. There are two leading accounts to explain the upper limit of VWM capacity. According to the slot model, visual working memory has a discrete limit on the number of items it can retain, or a limited number of available working memory “slots”. When all slots are filled, no information about additional items is maintained [11], [15], [16], [20], [26]. Thus, CDA should increase as more slots are utilized, regardless of the representation resolution. Therefore, in both conditions CDA amplitudes should reflect a fixed upper limit in capacity. According to the resource model, working memory capacity relies on dynamic resources [19], [18], [42], which can be allocated flexibly to accommodate increasing representation resolution, albeit with less upper limit in terms of capacity. Thus, there should be trade-offs between capacity upper limit and representation resolution, and the capacity upper limit reflected by CDA amplitude should be higher in low-resolution than in the high-resolution condition.

Although previous ERP results using orientation as the memory stimuli largely support the resource model [36], [37], Ikkai et al. [39], Luria et al. [41] and our results reject the resource model and provide strong evidence for the slot model. In particular, our results on the asymptote of CDA amplitude stand in contrast to Machizawa et al.’s findings [36], which was obtained under a very similar experimental paradigm. One salient difference between these two sets of studies, Ikkai et al. [39], Luria et al. [41] and our study vs. Machizawa et al. [36] and Gao et al. [37], is the visual stimuli used: whereas the latter studies used boundary objects and oriented stimuli, we and others (Ikkai et al. [39] and Luria et al. [41]) used colors (a surface feature). It is possible that there are genuine differences in processing mechanisms for color and orientation features in VWM. Many recent behavioral studies have shown different performance for color and orientation information in working memory tasks. Stevanovski et al. [43] found that memory in the color condition was significantly better than memory in the orientation condition. Woodman et al. [29] argued that the performance difference for color versus orientation was due to different consolidation rates in VWM, with a faster rate for color than for orientation. Another series of study examining the bandwidth of consolidation also found differences between color and orientation information [44]–[46]. In particular, consolidation of orientation into VWM is a serial process but the consolidation of color into VWM can occur in parallel for at least two colors. Finally, Alvarez et al. [47] found that there are different codes for objects defined by boundary feature and objects defined by surface features, and that shapes or orientations belong to boundary objects and colors belong to surface objects. Thus, our finding on VWM capacity with color stimuli, consistent with findings using color stimuli but inconsistent with findings using orientation stimuli [36], [37], [39], [41], is plausible given different processing mechanisms for color and orientation information in VWM.

Together, our results and previous results suggest that the relationship between VWM capacity and representation resolution varies for different visual features. For orientation features, capacity and resolution can exhibit trade-off, whereas for color, there is a fixed capacity regardless of resolution demand. Our results caution against generalization of results across visual features in studies of VWM. Future research is needed to probe the mechanisms responsible for the observed difference between color and other features and also to more systematically investigate VWM capacity and resolution for different visual materials.
